# 782. Employing the 4MAT model of instruction to develop a novel interactive virtual geriatrics course for interprofessional HIV clinicians

**DOI:** 10.1093/ofid/ofad500.843

**Published:** 2023-11-27

**Authors:** Aroonsiri Howell, Amelia Ross, Noelle Marie Javier, Jonathan Appelbaum, Laurie L Dozier

**Affiliations:** Temple University Hospital, Philadelphia, Pennsylvania; Lewis Katz School of Medicine at Temple University, Philadelphia, Pennsylvania; Icahn School of Medicine at Mt. Sinai, New York, New York; Florida State University College of Medicine, Tallahassee, Florida

## Abstract

**Background:**

With the advent of antiretroviral therapy, people with HIV (PWH) are living longer and experiencing geriatric syndromes. Many older PWH are cared for by teams of interprofessional HIV clinicians, most of whom lack geriatrics training. We aimed to increase geriatrics knowledge and preparedness to care for older PWH among interprofessional HIV clinicians through a unique application of the 4MAT model.

**Methods:**

From July 2020-August 2021, we enrolled physicians, nurse practitioners, physician assistants, and pharmacists in a virtual 7-hour course. Learners were assessed by pre- and post-course quizzes and self-reported preparedness to care for older PWH. There were 75 learners (44% physicians, 31% nurse practitioners/physician assistants, 24% pharmacists).

**Figure:**
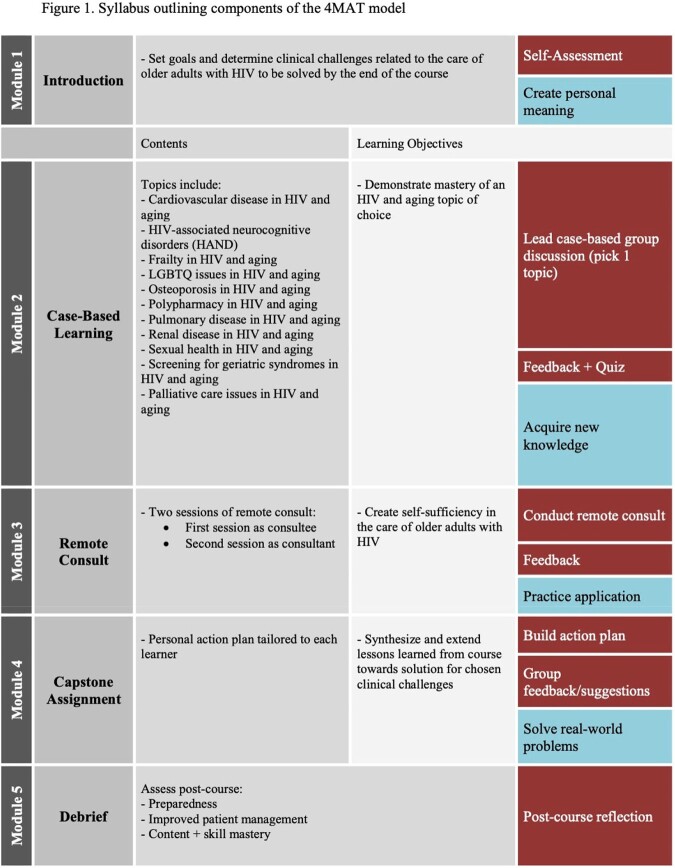

**Results:**

Before the course, 49.3% of learners felt prepared to care for older PWH vs. 93.7% post-course (p< 0.00). Average quiz scores improved on all topics (p< 0.00).

**Figure:**
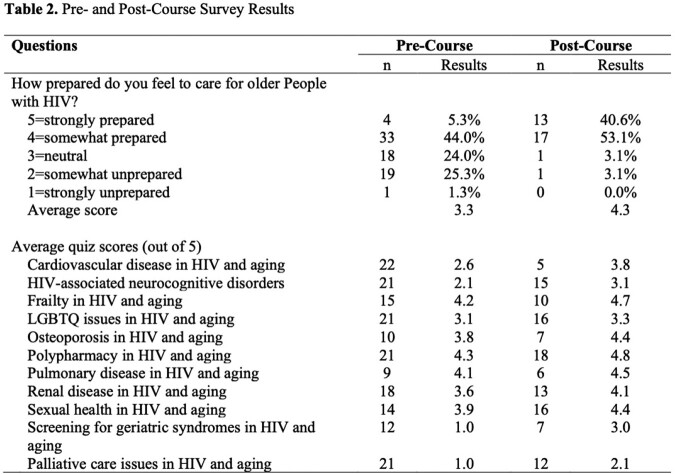

**Conclusion:**

Our course is a unique application of the 4MAT model that improved geriatrics knowledge and clinical preparedness across a wide array of interprofessional HIV clinicians.

**Disclosures:**

**All Authors**: No reported disclosures

